# miRNA Processing: Dicer-1 Meets Its Match

**DOI:** 10.1371/journal.pbio.0030244

**Published:** 2005-05-24

In recent years, the control of gene expression by small RNA molecules has emerged as a major new mechanism for gene regulation. The small RNAs interfere with the expression of their target gene by reducing its transcription, triggering the destruction of the gene transcript, or inhibiting its translation into a protein. This discovery has not only altered views of gene regulation, but also provided molecular geneticists with powerful new tools with which to study and manipulate the function of any gene. The biology of these small RNAs is, therefore, under intense scrutiny.

Small RNAs are generated by specific pathways, the elements of which are being rapidly discovered. In this issue of *PLoS Biology*, two groups have identified a missing piece in one such pathway—in the fruitfly Drosophila. The pathway under investigation leads to the production of a type of small RNA called a microRNA (miRNA). These are 21–23 nucleotides in length, and are involved in regulating the expression of many genes. miRNAs start life as a much bigger transcript called a pri-miRNA, which is processed in two steps. First, it is converted into a shorter pre-miRNA, by the action of two proteins: Drosha, an RNAse III enzyme; and Pasha, which contains double-stranded RNA binding domains (dsRBDs). The pre-miRNA is then transported to the cytoplasm and is trimmed again into a double-stranded miRNA by a different RNAse III enzyme called Dicer-1.

In a separate pathway, RNAs called small interfering RNAs (siRNAs) depend on the Dicer-2 RNAse III and a dsRBD protein called R2D2 for their function. These pathways are also conserved in other organisms. Thus, a pattern emerges: the functions of small RNAs tend to require the combined actions of an RNAse III and a dsRBD protein. But why doesn't Dicer-1 have a partner? The answer, provided by the two studies from the labs of Phil Zamore and Haruhiko and Mikiko Siomi, is that we just hadn't found it yet.

The two groups took different approaches to finding Dicer-1's partner. Zamore's group looked for genes resembling other dsRBD-encoding genes, while the Siomi lab did a functional screen for new genes specifically implicated in miRNA processing. They both homed in on a new gene with great similarity to R2D2, and showed that loss of function of the gene results in the accumulation of pre-miRNAs—very similar to loss of Dicer-1 function, which suggests that the two genes act together in the same pathway. The new potential partner of Dicer-1 was given the name *loquacious (loqs)*, because failure to process the miRNAs in turn causes increased levels of expression of the target genes for the miRNAs.

Both groups also show that Loqs and Dicer-1 exist in a complex within the cell, and that the complex is able to process pre-miRNA into its mature form. The Siomis' lab went on to show that the complex contains a protein called Ago-1, which hints that the complex might also be involved in the action of miRNAs on their target genes, as well as in miRNA processing itself. Both groups also point out the similarity between Loqs and a human dsRBD protein called TRBP, which has been implicated in the response to infection by HIV.

There seems little doubt, then, that Dicer-1's partner has been found, and that the combined action of an RNAse III and a dsRBD protein is a consistent theme in the function of miRNAs and siRNAs. The identification of Loqs will help to refine our views of how miRNAs are processed, as well as how they can be manipulated. The connections made with processes such as stem cell maintenance (identified by the Zamore lab) and viral infection in these new studies also emphasize that gene regulation by small RNAs is relevant to a broad range of cellular physiology.

